# The small molecule WNT/β-catenin inhibitor CWP232291 blocks the growth of castration-resistant prostate cancer by activating the endoplasmic reticulum stress pathway

**DOI:** 10.1186/s13046-019-1342-5

**Published:** 2019-08-06

**Authors:** Sahyun Pak, Sejun Park, Yunlim Kim, Jung-Hyuck Park, Chan-Hee Park, Kyoung-June Lee, Choung-soo Kim, Hanjong Ahn

**Affiliations:** 1 Department of Urology, Center for Urologic Cancer, National Cancer Center, Goyang, South Korea; 2 Department of Urology, University of Ulsan College of Medicine, Ulsan University Hospital, Ulsan, South Korea; 3 Department of Urology, University of Ulsan College of Medicine, Asan Medical Center, Seoul, South Korea; 4 Asan Institute for Life Science, Asan Medical Center, Seoul, South Korea; 5 Drug Discovery Center, JW Pharmaceutical Corporation, Seoul, South Korea; 6 JW Creagene Corporation, Seongnam, South Korea

**Keywords:** Prostate cancer, Castration-resistant, WNT signaling pathway, Endoplasmic reticulum stress

## Abstract

**Background:**

Androgen receptor (AR)-targeted treatments improve the survival of castration-resistant prostate cancer (CRPC) patients; however, secondary resistance to these agents ultimately occurs in virtually all patients. Therefore, alternative therapeutic targets are urgently needed. Since growing evidence demonstrates that WNT/β-catenin signaling plays an important role in CRPC, the antitumor activity and mechanism of action of CWP232291, a small molecule β-catenin inhibitor, were investigated in prostate cancer.

**Methods:**

We assessed the antitumor activity of CWP232291 in prostate cancer cell lines and primary cells derived from CRPC patients. The effect of CWP232291 on apoptotic cell death, endoplasmic reticulum (ER) stress, cell viability, and WNT/β-catenin signaling was evaluated by flow cytometry, western blotting, luciferase reporter assay, and fluorescence microscopy. Antitumor efficacy was assessed in two CRPC xenograft mouse models.

**Results:**

CWP232291 induced ER stress, resulting in upregulation of the proapoptotic protein CHOP and activation of caspase-3-dependent apoptosis. In addition, CWP232291 suppressed the expression of β-catenin by affecting WNT-dependent transcriptional activity, and downregulated AR and its splice variants in prostate cancer cells. Antitumor activity was observed in prostate cancer cells in vitro and ex vivo, and antitumor efficacy was observed in vivo.

**Conclusions:**

Beyond providing preclinical evidence of therapeutic efficacy for the novel small molecule β-catenin inhibitor CWP232291 in CRPC, our results show that inducing ER stress and targeting WNT/β-catenin signaling may be a novel strategy against CRPC.

**Electronic supplementary material:**

The online version of this article (10.1186/s13046-019-1342-5) contains supplementary material, which is available to authorized users.

## Background

Metastatic prostate cancer initially responds to androgen deprivation therapy (ADT) [1, 2]. Unfortunately, nearly all patients with metastatic prostate cancer eventually develop resistance to ADT, a state called castration-resistant prostate cancer (CRPC). Prostate cancer deaths are typically the result of metastatic CRPC, and the median survival for men with CRPC is less than 2 years [3].

Androgen receptor (AR)-dependent mechanisms represent the main pathway through which CRPC develops. These mechanisms include amplification and/or AR mutation, AR splice variant expression, increased production of androgen, and changes in the activity or expression of AR co-activators and co-repressors [4]. Although AR-targeted second-generation ADT treatments such as abiraterone [1] and enzalutamide [2] improve the survival of CRPC patients, secondary resistance to these agents ultimately occurs in virtually all patients [5]. Therefore, alternative targets and novel therapies are urgently needed.

WNT/β-catenin signaling is an evolutionarily conserved pathway that plays a role in cellular proliferation, differentiation, and migration in multiple organ systems [6]. WNT activation can induce two different pathways, the β-catenin-dependent canonical pathway and the β-catenin-independent non-canonical pathway. In the absence of WNT ligands, β-catenin is recruited and degraded by the destruction complex. Binding of WNT to its receptors disrupts the destruction complex, thereby inducing cytoplasmic accumulation of β-catenin and subsequent translocation to the nucleus.

Aberrations within the WNT/β-catenin pathway are implicated in many forms of human disease including cancer [7]. Growing evidence demonstrates that the WNT/β-catenin signaling pathway also plays an important role in CRPC. For example, significantly mutated WNT signaling was identified in lethal CRPC [8]. The WNT pathway acts as a key regulator by integrating signals from the PI3K/mTOR, MAPK, and AR pathways [9]. Importantly, crosstalk between β-catenin and AR signaling in CRPC has been amply documented [10–16]. Nuclear co-localization and interaction of endogenous AR with β-catenin are more frequently observed in CRPC than in hormone-naïve prostate cancer. Although targeting the WNT/β-catenin pathway is considered a promising approach in CRPC, the therapeutic efficacy of WNT/β-catenin pathway modulators in prostate cancer remains largely unknown.

CWP232291 (U.S. Patent 8,940,739 B2), a novel peptidomimetic small molecule, induces tumor-selective apoptosis and modulates the WNT/β-catenin pathway [17]. CWP232291 is currently being tested in phase 1 trials for hematological cancers as a potent β-catenin inhibitor [17–19]. Although preclinical studies reported that CWP232228, which is closely related to CWP232291, inhibits the transcriptional activity of β-catenin [20, 21], the molecular mechanism via which CWP232291 induces apoptotic cell death has not been fully elucidated.

This study investigated the mechanism by which CWP232291 induces apoptosis and the effect of CWP232291 on WNT/β-catenin and AR signaling in prostate cancer cells. The antitumor activity of CWP232291 in prostate cancer was assessed in vitro, ex vivo, and in vivo.

## Materials and methods

### Reagents and antibodies

CWP232291 was obtained from JW Pharmaceutical Corporation (Seoul, Korea). The compound z-valine-alanine-aspartate-fluoromethylketone (ZVAD-FMK) was purchased from R&D Systems (Minneapolis, MN). The following primary antibodies were used: cleaved caspase-3, poly (ADP-ribose) polymerase (PARP), phospho-eIF2a serine 51 [peIF2a (serine-51)], eIF2a, inositol-requiring kinase 1 (IRE1), C/EBP-homologous protein (CHOP) (Cell Signaling Technology, Danvers, MA), AR, survivin, GAPDH, β-actin, bcl-2 (Santa Cruz Biotechnology, Dallas, TX), β-catenin (Merck Millipore, Burlington, MA) and AR-V7 (Precision Antibody, Columbia, MD).

### Cell culture

The human prostate cancer cell lines PC3, DU145, VCaP, LNCaP, and 22Rv1 were obtained from the American Type Culture Collection (ATCC; Manassas, VA) and maintained in RPMI 1640 (Invitrogen, Waltham, MA) with 5–10% heat-inactivated fetal bovine serum (FBS), 100 units/mL penicillin, and 100 μg/mL streptomycin in a 5% CO_2_ atmosphere at 37 °C. After informed consent, prostate cancer tissues were obtained from patients who underwent palliative transurethral resection of the prostate to relieve bladder outlet obstruction and were subsequently diagnosed with prostate cancer at Asan Medical Center. The study protocol was approved by the Institutional Review Board of Asan Medical Center. Tumor specimens were minced with scissors and digested by incubation in RPMI containing 1 mg/mL type I collagenase (Sigma Aldrich, St. Louis, MO) for 1 h at 37 °C. Cells were washed with medium containing 10% FBS to inactivate collagenase and then with PBS to remove FBS. Next, cells were plated and maintained in Human Prostate Epithelial Cell Growth Medium (Lonza, Portsmouth, NH) in a 5% CO_2_ atmosphere at 37 °C. To ensure authentication and consistency throughout the study, only low-passage cells (< passage 8–15) were used in the experiments. Mycoplasma testing was performed using a PCR-based e-mycoplasma test kit (iNtRON Biotechnology, Seongnam, Korea) for all cells.

### Cell viability assay

Cell viability was measured using the CellTiter Glo® cell viability assay (Promega, Madison, WI). Briefly, 3 × 10^3^ cells were seeded per well in 96-well plates and incubated overnight. After exposure to CWP232291, cells were incubated with 20 μL of CellTiter Glo reagent for 10 min, after which luminescence intensity was measured on a MicroLumatPlus LB luminometer (EG&G Berthold, Bad Wildbad, Germany). All plates had blank wells containing cell-free medium to measure background luminescence. Data represent the percentage of control untreated cells [(treatment value − blank) / (vehicle value − blank)] expressed as the mean ± SD of at least three repetitions. Results were analyzed using the GraphPad Prism® version 6 software. Drug concentrations that reduced response to 50% of control (IC_50_) were determined for each cell line.

### Lactate dehydrogenase cytotoxicity assay

The cell cytotoxicity assay was performed using the Cytotoxicity Detection Kit (Sigma Aldrich, St. Louis, MO) according to the manufacturer’s protocol. Briefly, 3 × 103 cells were seeded per well into 96-well plates and incubated overnight. After exposure to CWP232291, 50 μL of sample medium (from control and concentration dependent treatments) were transferred to a 96-well plate in triplicate wells. Reaction mixture (50 μL) was then added to each sample and incubated for 30 min in the dark at room temperature. The reaction was stopped by adding 50 μL of stop solution and mixing by gentle tapping. The absorbance was measured at 492 nm and 680 nm. The 680n m absorbance value (background) was subtracted from the 492 nm absorbance values before calculation of % cytotoxicity. The maximum lactate dehydrogenase (LDH) activity was determined after treating cell with lysis buffer as described in the manufacturer’s protocol. Cytotoxicity (%) was calculated using the following formula:

Cytotoxicity (%) = (CWP-treated LDH activity-Low LDH activity) / (High LDH activity-Low LDH activity) X 100.

### Western blot analysis

Whole cell lysates were prepared in lysis buffer [150 mmol/L NaCl, 1% Nonidet P-40, 50 mmol/L Tris–HCl (pH 7.4), 50 mmol/L NaF, 5 mmol/L EDTA, 0.1 mmol/L Na_3_VO_4_, and 0.1% SDS] containing protease inhibitor cocktail (Sigma Aldrich). The cell lysates were microcentrifuged at 13,000×g for 10 min, and the supernatants were stored at − 80 °C. Protein concentration was measured using the Bradford protein assay (Bio-Rad, Hercules, CA). Proteins were separated by electrophoresis and transferred to PVDF membrane. After blocking with 5% bovine serum albumin (BSA) for 1 h at room temperature, the membranes were incubated with primary antibody, then with secondary antibody conjugated with peroxidase. Protein bands were detected using the chemiluminescence detection system (Millipore Corp., Billerica, MA).

### RNA extraction and quantitative RT-PCR analysis

Total RNA was extracted from untreated or CWP232291-treated cell lines using Trizol® (Invitrogen) according to the manufacturer’s instructions. The DNase-treated RNA was reverse-transcribed using a cDNA synthesis kit (Toyobo, Osaka, Japan). Quantitative PCR was conducted using the SYBR method (Toyobo). The PCR thermal cycling conditions were as follows: 95 °C for 20 s, followed by 40 cycles of 95 °C for 3 s, and 60 °C for 30 s. The melting curve stage proceeded at 95 °C for 15 s, melting from 60 °C for 1 min to 95 °C for 15 s with a ramp rate of 1%, and 60 °C for 15 s. Melting curve analysis was performed to ensure the specificity of the PCR products. GAPDH was selected for internal reference and loading control. The following primers were used: CHOP forward, 5′-AGAACCAGGAAACGGAAACAGA-3′, reverse, 5′-TCTCCTTCATGCGCTGCTTT-3′; AR forward, 5′-CAGTGGATGGGCTGAAAAAT-3′, reverse 5′- AAGCGTCTTGAGCAGGATGT-3′; PSA forward, 5′-CATCAGGAACAAAAGCGTGA-3′, reverse, 5′-ATATCGTAGAGCGGGTGTGG-3′; UBE2C forward, 5′-AGTGGCTACCCTTACAATGCG-3′, reverse, 5′-TTACCCTGGGTGTCCACGTT-3′; UGT2B17 forward, 5′- ACCAGCCAAACCCTTGCCTAAG-3′, reverse, 5′-GGCTGATGCAATCATGTTGGCAC-3′; TMPSS2 forward, 5′-CAGGAGTGTACGGGAATGTGATGGT-3′, reverse, 5′-GATTAGCCGTCTGCCCTCATTTGT-3′; c-myc forward, 5′- GCTGCTTAGACGCTGGATTT-3′, reverse, 5′- GGCATTCGACTCATCTCAGC-3′; cyclin D1 forward, 5′- ATGTTCGTGGCCTCTAAGATGA-3′, reverse, 5′- CCAGTGGTTACCAGCAGCTC-3′; MMP-7 forward, 5′- TGAGCTACAGTGGGAACAGG-3′, reverse, 5′- ACCACCCCAAAGAAAATTCC-3′; Annexin-2 forward, 5′-ACGCTGGAGTGAAGAGGAAA-3′, reverse, 5′- AAGGCACTGAGACTCCCTCA-3′; Axin-2 forward, 5′- AGTGTGAGGTCCACGGAAAC-3′, reverse, 5′- TGGCTGGTGCAAAGACATAG-3′; and GAPDH forward, 5′-CAATGACCCCTTCATTGACC-3′, reverse, 5′-GACAAGCTTCCCGTTCTCAG-3′.

### Immunofluorescence staining and confocal microscopy

Cells were fixed with 4% paraformaldehyde in PBS for 20 min and permeabilized with 0.1% Triton X-100 in PBS for 30 min. Non-specific binding sites were blocked with 5% BSA in PBS for 1 h, then cells were incubated overnight at 4 °C with primary antibody against AR followed by secondary antibody conjugated with fluorescent dye (Molecular Probes, Eugene, OR). The samples were mounted in Vectashield medium containing 4′,6-diamidino-2-phenylindole (DAPI) (Vector Laboratories, Burlingame, CA). Confocal images were obtained using a confocal laser microscopy system (Leica Geosystems, Heerbrugg, Switzerland).

### Transient transfection and dual luciferase reporter assay

The TCF/LEF reporter construct is a mixture of an inducible β-catenin-responsive luciferase construct and a constitutively expressed Renilla element. The β-catenin-responsive luciferase construct encodes the firefly luciferase reporter gene under the control of a minimal (m) CMV promoter and tandem repeats of the TCF/LEF transcriptional response element. For transfection, cells were seeded at 3 × 10^5^ cells/well (PC3, DU145) and 6 × 10^5^ cells/well (LNCaP, 22Rv1) in 6-well plates. After 18–24 h, cells were transfected with 1 μg of TCF/LEF reporter construct (Qiagen GmbH, Hilden, Germany) using 2 μL of Lipofectamine 2000™ (Invitrogen) in 50 μL of OptiMEM® reduced-serum medium, achieving a DNA/Lipofectamine 2000™ ratio of 1:2. After transfection, cells were treated with or without 100 ng/mL WNT3A (R&D Systems) and CWP232291 for 24 h; control cells were untreated. Luciferase assays were performed using the Dual-Glo luciferase assay system (Promega). Each assay was performed in triplicates, and the reporter activity was expressed as mean ± SD.

### Apoptosis assay (annexin V staining)

Cells were seeded in 6-well plates in RPMI 1640 medium containing 10% FBS for 18–24 h. The cells were then exposed to CWP232291 for 72 h, after which apoptosis and necrosis were assessed by flow cytometry using the annexin V-FITC apoptosis detection assay (BD Biosciences, Bedford, MA) in accordance with the manufacturer’s instructions. In this assay, cells positive for annexin V (bottom right quadrant) and those positive for both annexin V and propidium iodide (PI) (top right quadrant) represent the early and late apoptotic populations, respectively, whereas cells positive for PI only (top left quadrant) represent the necrotic population. The apoptosis cell population analysis was carried out using CellQuestPro software (BD Biosciences).

### Tumor xenograft models

The experiments were approved by the Institutional Animal Care and Use Committee (IACUC) of Asan Medical Center (2015–14-182). 22Rv1 cells (5 × 10^6^) were injected subcutaneously into the right dorsal flanks of 6-week-old male BALB/C nude mice (OrientBio, Seoul, Korea). When the tumors reached an average volume of 70–100 mm^3^, the mice were randomly divided into control and treatment groups. Mice were treated with CWP232291 in 3% dimethyl sulfoxide in phosphate buffered saline, and control groups received an equal volume of the corresponding diluent alone. 22Rv1-bearing mice received intravenous (tail vein) CWP232291 (50 or 100 mg/kg/day) or vehicle alone once per week for 4 weeks (*n* = 6 per group). The tumor volume was measured three times a week and calculated using the following formula: 0.52 × length × (width)^2^ (length: longest diameter across the tumor; width: corresponding perpendicular diameter). The concentration-time profile of the active metabolite (CWP232204) after a single intravenous injection of CWP232291 into nude mice is shown in Additional file 1: Figure S1, and CWP232291 pharmacokinetic variables are shown in Additional file 1: Table S1.

### Statistical analysis

Data were obtained from at least three independent experiments and presented as means ± SD. Statistical evaluation of the results was performed by one-way and two-way ANOVA with Dunnett multiple comparisons test. *P* values < 0.05 were considered statistically significant. Prism 6 (GraphPad Software) was used to calculate and analyze the statistical differences between experimental groups.

### CWP232291 induces apoptosis in prostate cancer cells

**Fig. 1:**
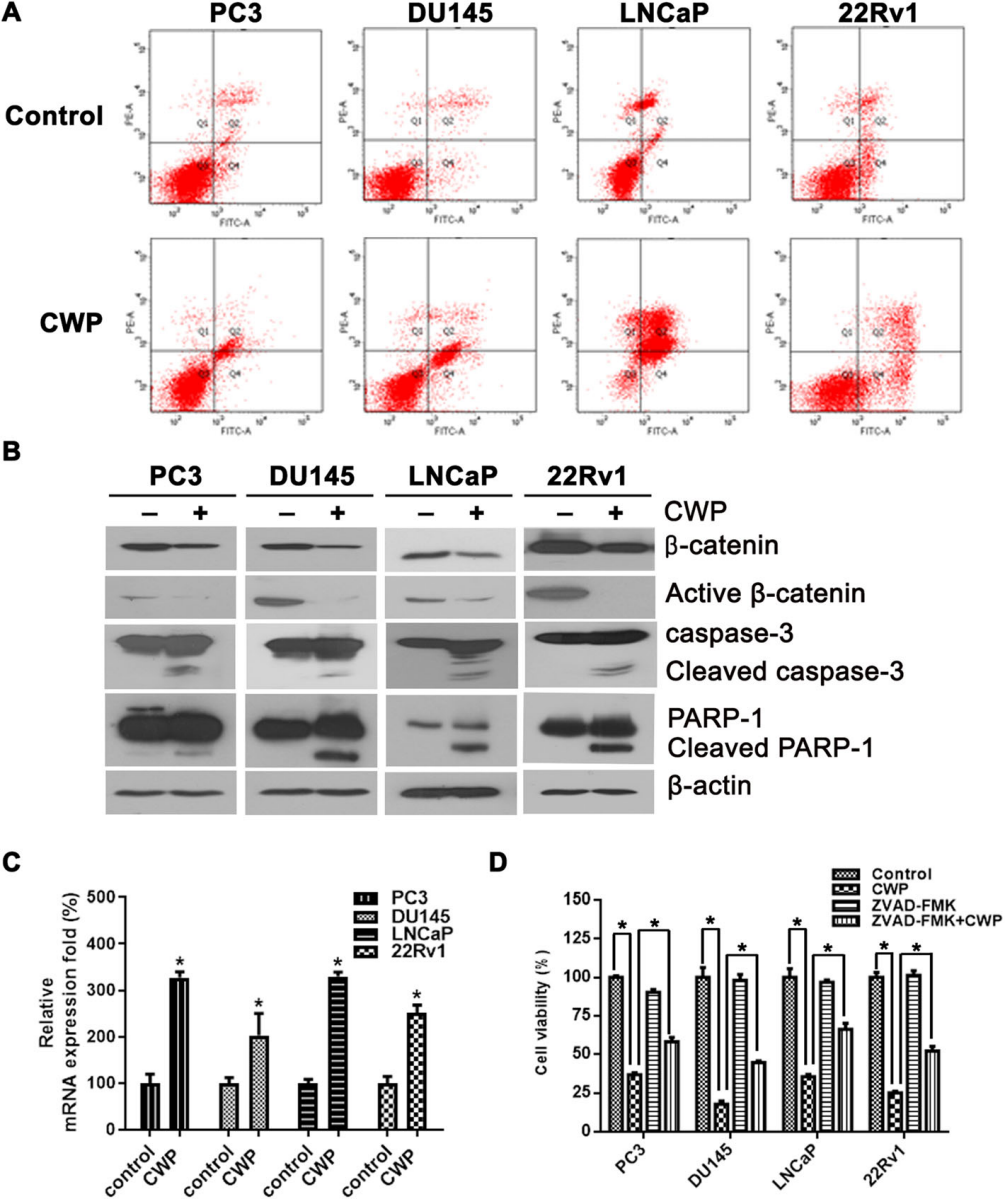
CWP232291 induces apoptosis and cell cycle arrest in prostate cancer cells. **a** Cells were exposed to CWP232291 for 72 h at the IC_50_. Annexin V/PI-stained cells were assessed by flow cytometry. The presence of annexin V-positive and PI-negative cells showed apoptotic cell death, and the presence of annexin V-positive and PI-positive cells showed late apoptosis and necrotic cell death. **b** Cells were exposed to CWP232291 for 72 h at the IC_50_. Changes in caspase, PARP-1, and indicated proteins were analyzed by western blotting. **c** Cells were exposed to IC_50_ doses of CWP232291 for 24 h (PC3, 200 nM; DU145, 400 nM; LNCaP, 60 nM; 22Rv1, 70 nM). Relative Axin-2 mRNA levels were quantified by real-time PCR in prostate cancer cells (means ± SD, *n* = 3, *P < 0.05 compared with the untreated control). **d** Cells were exposed to CWP232291 for 96 h at the IC_50_ (PC3, 200 nM; DU145, 400 nM; LNCaP, 60 nM; 22Rv1, 70 nM) in the presence or absence of 10 μmol/L Z-VAD-FMK. Cell viability was expressed as the means ± SD of three independent experiments. *P < 0.05 by one-way ANOVA

First, we investigated whether CWP232291 induces apoptosis in prostate cancer cells. We selected four prostate cancer cells: PC3 and DU145 (AR-negative and androgen-independent), LNCaP (AR-expressing and androgen-dependent), and 22Rv1 (AR-expressing and androgen-independent). Flow cytometry analysis of prostate cancer cells doubly labeled with annexin V and PI showed that CWP232291 induced apoptosis (Fig. 1a). In addition, exposure of cells for 72 h to a concentration of CWP232291 equivalent to the IC_50_ (PC3, 200 nM; DU145, 400 nM; LNCaP, 60 nM; 22Rv1, 70 nM) significantly increased apoptosis compared with the control in PC3, DU145, LNCaP, and 22Rv1 cells. At the same time point, cleaved caspase-3 and cleaved PARP were significantly increased by CWP232291 compared with the control (Fig. 1b). mRNA levels of Axin-2, a negative regulator of canonical WNT signaling, was increased in prostate cancer cells after treatment with IC_50_ doses of CWP232291 (Fig. 1c). Inhibition of caspase activity with ZVAD-FMK, a cell-permeable pan-caspase inhibitor, abrogated CWP232291-induced apoptosis, demonstrating that CWP232291 kills cells through the caspase pathway (Fig. 1d).

### CWP232291 induces endoplasmic reticulum stress and the unfolded protein response

**Fig. 2:**
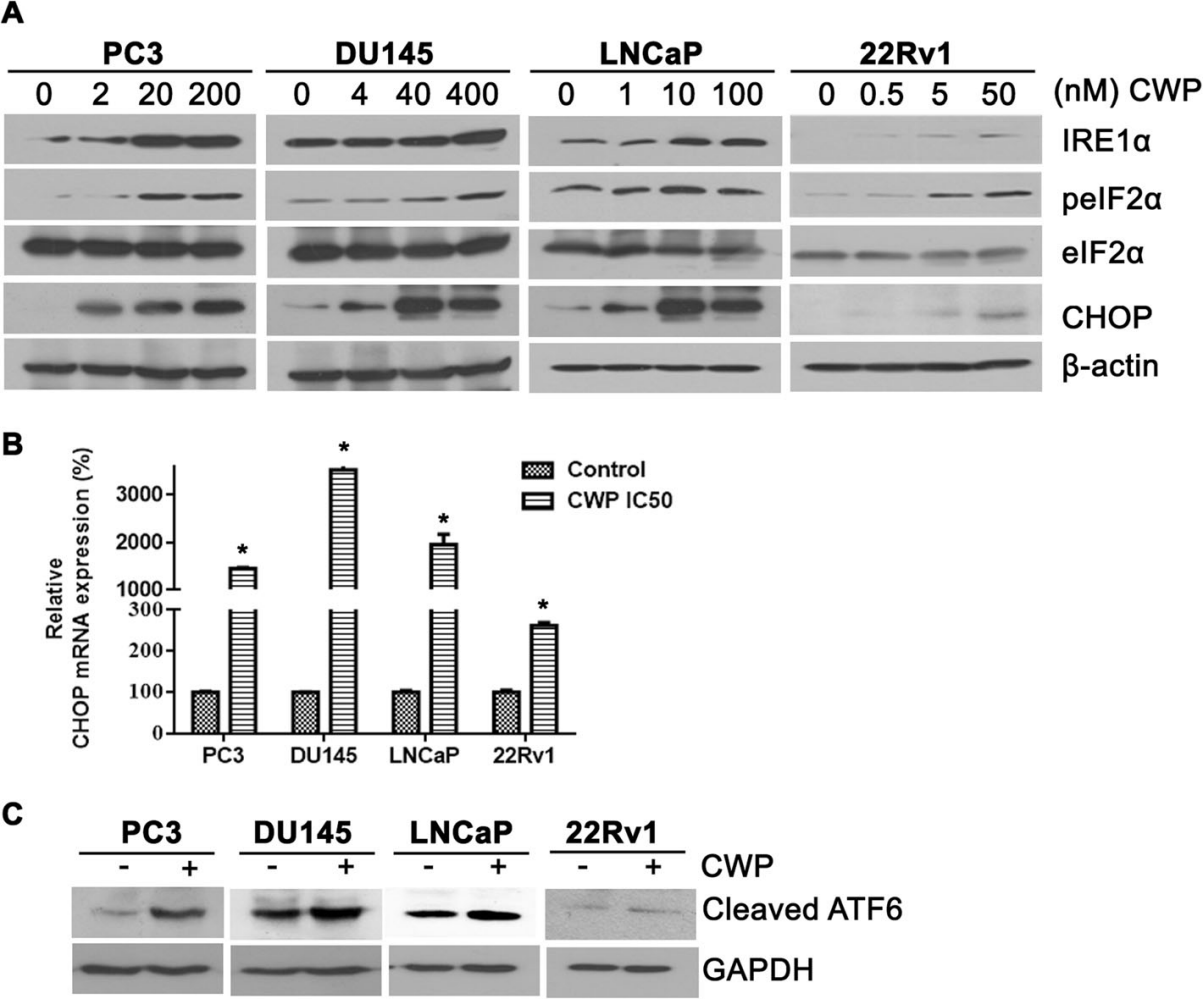
CWP232291 induces endoplasmic reticulum stress and the unfolded protein response in prostate cancer cells. **a** Cells were exposed to CWP232291 for 24 h. Western blot analysis was performed using IRE1α, phospho-eIF2α, eIF2α, and CHOP antibodies. Actin was used as a loading control. **b** Cells were exposed to CWP232291 for 24 h at the IC_50_ (PC3, 200 nM; DU145, 400 nM; LNCaP, 60 nM; 22Rv1, 70 nM). Relative CHOP mRNA levels were quantified by real-time PCR in prostate cancer cells (means ± SD, *n* = 3, *P < 0.05 compared with the untreated control). **c** Cells were exposed to CWP232291 for 24 h at the IC_50_ (PC3, 200 nM; DU145, 400 nM; LNCaP, 60 nM; 22Rv1, 70 nM). Cleaved ATF6 protein was analyzed by western blotting

Next, we investigated whether CWP232291 induces endoplasmic reticulum (ER) stress and the unfolded protein response (UPR). ER stress triggers the UPR, which is mediated by three sensors: protein kinase RNA-like ER kinase (PERK), inositol-requiring enzyme 1 (IRE1), and activating transcription factor 6 (ATF6) [22]. PERK activation attenuates global translation initiation via phosphorylation of eukaryotic translation initiation factor 2α (eIF2α), resulting in increased expression of the proapoptotic CHOP [23]. Cells were exposed to CWP232291 for 24 h at the IC_50_ (PC3, 200 nM; DU145, 400 nM; LNCaP, 60 nM; 22Rv1, 70 nM), after which ER stress-related markers were assessed by western blotting and real-time PCR (Fig. 2). A concentration-dependent increase in eIF2α protein phosphorylation, IRE1 protein, and CHOP protein and mRNA was observed, suggesting that CWP232291 induces ER stress. Taken together, these data indicate that CWP232291 induces ER stress, resulting in PERK activation, upregulation of CHOP, and caspase-3-dependent apoptosis.

### CWP232291 inhibits expression of β-catenin in prostate cancer cells

**Fig. 3:**
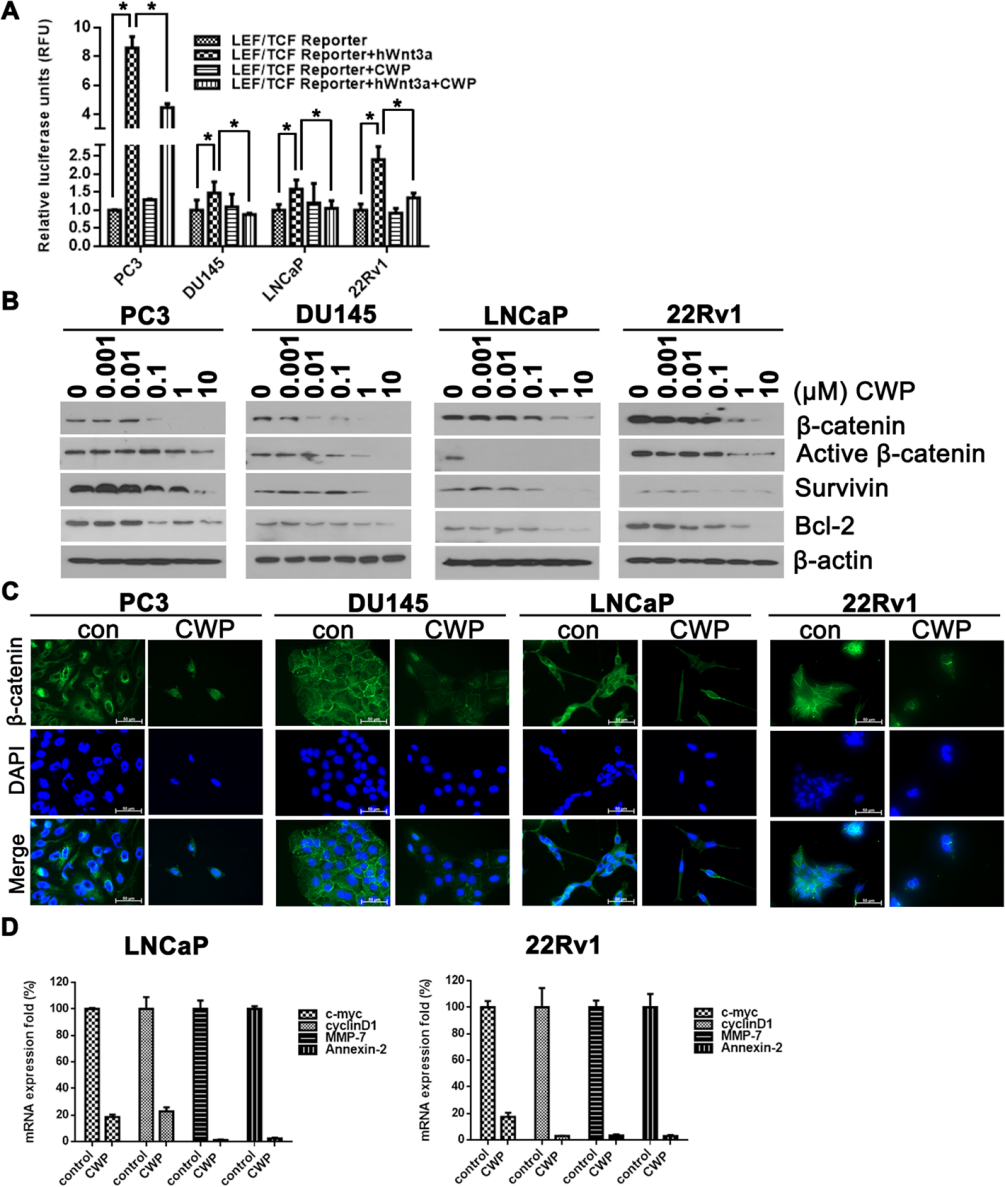
CWP232291 inhibits the expression of β-catenin and survivin in prostate cancer cells. **a** Reporter assay for WNT/β-catenin signaling after treatment with (+) or without (−) WNT3a (100 ng/mL) and CWP232291 (PC3, 200 nM; DU145, 400 nM; LNCaP, 60 nM; 22Rv1, 70 nM) in cell cultures. Results are expressed as means ± SD of three independent experiments. *P < 0.05 by one-way ANOVA. **b** Cells were exposed to CWP232291 for 24 h. Lysates were analyzed by western blotting with β-catenin, survivin, and bcl-2 antibodies. Actin was used as a loading control. Scale bar, 100 μm. **c** Cells were exposed to CWP232291 for 24 h and then stained with DAPI (blue) or β-catenin (green). Images were captured using a fluorescence microscope (Olympus). **d** LNCaP and (E) 22Rv1 cells were treated with or without IC_5_0 doses of CWP232291 for 24 h (LNCaP, 60 nM; 22Rv1, 70 nM). Relative mRNA levels of c-myc, cyclinD1, MMP-7, and annexin-2 were quantified by real-time PCR in prostate cancer cells

The targeting of WNT/β-catenin signaling by CWP232291 was investigated. In the canonical WNT pathway, LEF/TCF family members are the key mediators of β-catenin-dependent transcription [7, 24]. PC3, DU145, LNCaP, and 22Rv1 cells were transfected with a LEF/TCF luciferase reporter construct and exposed to CWP232291 (PC3, 200 nM; DU145, 400 nM; LNCaP, 60 nM; 22Rv1, 70 nM) in the presence or absence of WNT3a (100 ng/mL) (Fig. 3a). CWP232291 significantly decreased luciferase activity compared with the control, demonstrating inhibition of β-catenin and WNT target gene survivin. Indeed, western blotting revealed that CWP232291 markedly suppressed the expression of the β-catenin and survivin in prostate cancer cells in a concentration-dependent manner (Fig. 3b). In addition, by fluorescence microscopy, a decrease in the amount of β-catenin staining was observed in the nucleus of prostate cancer cells exposed to CWP232291 compared with the control. In the absence of CWP232291, β-catenin staining was diffuse and distributed throughout the cytoplasm. Treatment of prostate cancer cells with WNT3a increased the nuclear localization and expression of β-catenin (Additional file 1: Figure S2). β-catenin staining was lower in the nucleus of prostate cancer cells treated with CWP232291 than in the untreated control (Fig. 3c). Next, we investigated whether CWP232291 treatment reduces WNT/β-catenin target gene expression. mRNA levels of c-myc, cyclin D1, MMP-7, and annexin-2 were lower in LNCaP and 22Rv1 cells after treatment with IC_50_ doses of CWP232291 for 24 h (LNCaP, 60 nM; 22Rv1, 70 nM) than in the untreated control (Fig. 3d).

### CWP232291 downregulates AR and its splice variants

**Fig. 4:**
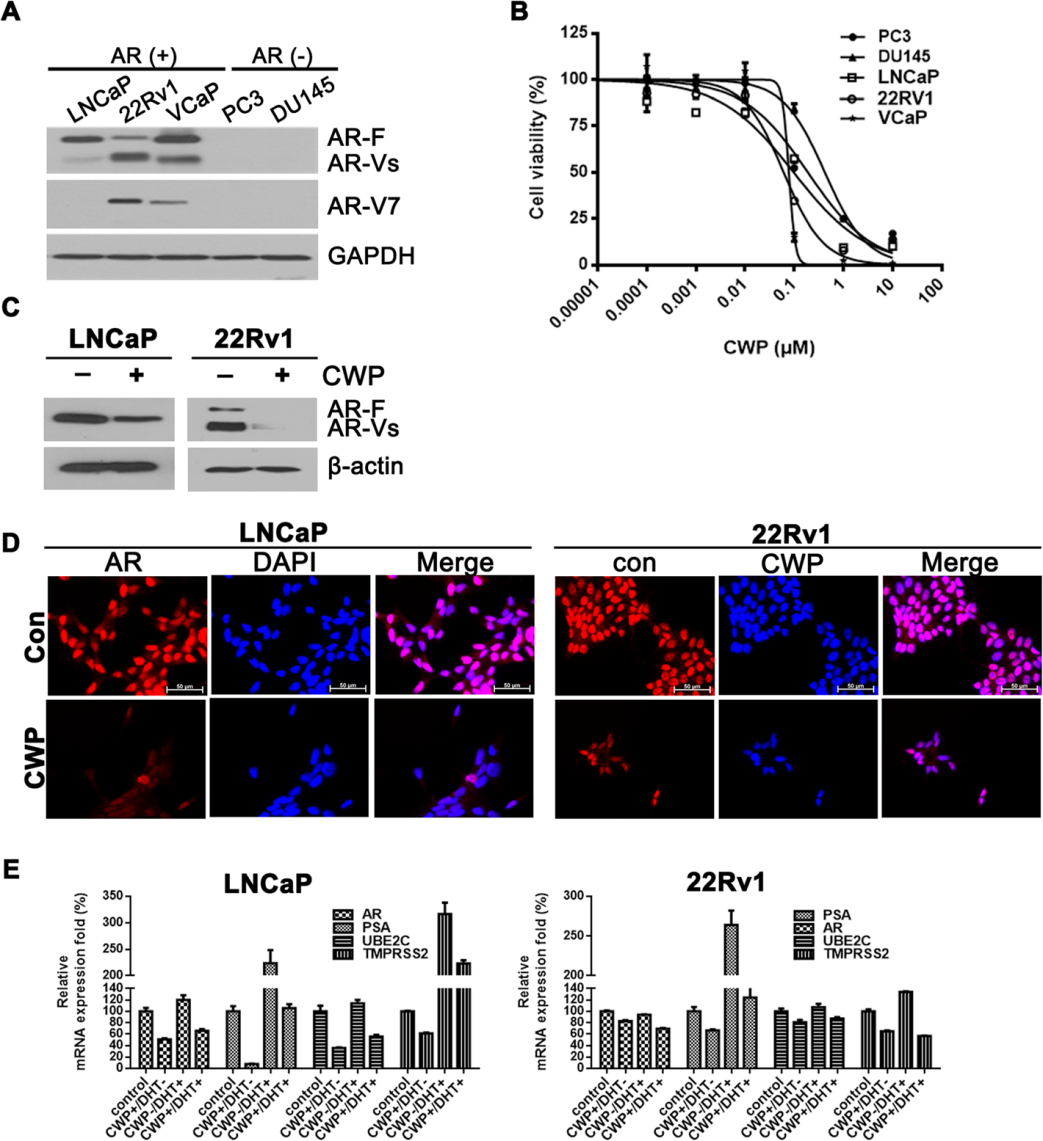
CWP232291 downregulates the androgen receptor and its splice variants. **a** AR status in prostate cancer cell lines. Western blot analysis was performed using AR, AR-Vs, and AR-V7 antibodies. GAPDH was used as a loading control. **b** Cells were exposed to 0–10 μM CWP232291 for 72 h. Cell viability was determined using CellTiter Glo® (means ± SD, *n* = 3). **c** LNCaP and 22Rv1 cells were exposed to CWP232291 for 72 h at the IC_50_. Western blotting was performed using AR antibodies. Actin was used as a loading control. **d** LNCaP and 22Rv1 cells were exposed to CWP232291 for 24 h at the IC_50_ and then stained with AR (red) and DAPI (blue). Images were captured using a fluorescence microscope. Scale bar, 100 μm. **e** LNCaP and (F) 22Rv1 cells were androgen-deprived for 48 h and then treated with vehicle or 1 nM DHT with or without IC_50_ doses of CWP (LNCaP 100 nM; 22Rv1 60 nM) for 24 h. Relative mRNA levels of AR, PSA, UBE2C, and TMPRSS2 were quantified by real-time PCR in prostate cancer cells

Given that β-catenin interacts with the AR, it is conceivable that CWP232291 may also affect AR activity. Prostate cancer cell lines were classified according to AR status: PC3 and DU145 (AR-negative), LNCaP (wild-type AR), 22Rv1 (AR splice variant), VCaP (AR overexpression). To investigate the effects of CWP232291 on AR, cell viability was assessed in AR-expressing (LNCaP, 22Rv1, and VCaP) and AR-negative (PC3 and DU145) prostate cancer cells (Fig. 4a). After prostate cancer cells were exposed to 0–10 μM CWP232291 for 72 h, cell viability was measured. CWP232291 reduced the proliferation of all prostate cancer cells. The IC_50_ values were lower in AR-expressing cells than in AR-negative cells (Fig. 4b; IC_50_ of 0.097 μM in LNCaP, 0.060 μM in 22Rv1, 0.070 μM in VCaP vs. 0.188 μM in PC3 and 0.418 μM in DU145 cells). The LDH cytotoxic assay was performed to investigate whether CWP232291 has cytotoxic effects in prostate cancer cells (Additional file 1: Figure S3). Released LDH, an indicator of cytotoxicity, increased at concentrations of CWP232291 higher than 0.01 μM. Western blotting showed that CWP232291 decreased the expression of AR and its splice variants in both LNCaP and 22Rv1 cells (Fig. 4c). Similarly, immunofluorescence staining and confocal microscopy revealed that CWP232291 markedly decreased AR expression in both LNCaP and 22Rv1 cells (Fig. 4)d.

Transcriptional regulation of AR by β-catenin through TCF/LEF-binding sites on the AR promoter has been well documented [10]. To investigate whether CWP232291 directly targets AR transcription, AR and its target gene mRNA levels were analyzed in LNCaP and 22Rv1 cells, which were androgen-deprived for 48 h and then treated with vehicle or 1 nM DHT with or without IC_50_ doses of CWP (LNCaP 100 nM; 22Rv1 60 nM) for 24 h. As shown in Fig. 4e, CWP232291 treatment decreased AR, PSA, UBE2C, and TMPRSS2 mRNA levels, suggesting that CWP232291 may directly target AR transcription. Additionally, we assessed the activity of androgen-response elements using a reporter assay. CWP232291 significantly suppressed androgen-response element luciferase activity in prostate cancer cells treated with DHT (Additional file 1: Figure S4). We performed the chromatin immunoprecipitation assay to confirm the transcriptional regulation of AR by β-catenin. The results revealed that CWP232291 inhibits β-catenin occupancy of the TCF-binding site within the AR promoter (Additional file 1: Figure S5).

### CWP232291 blocks the growth of androgen-independent prostate cancer cells and primary cells derived from CRPC patients

**Fig. 5:**
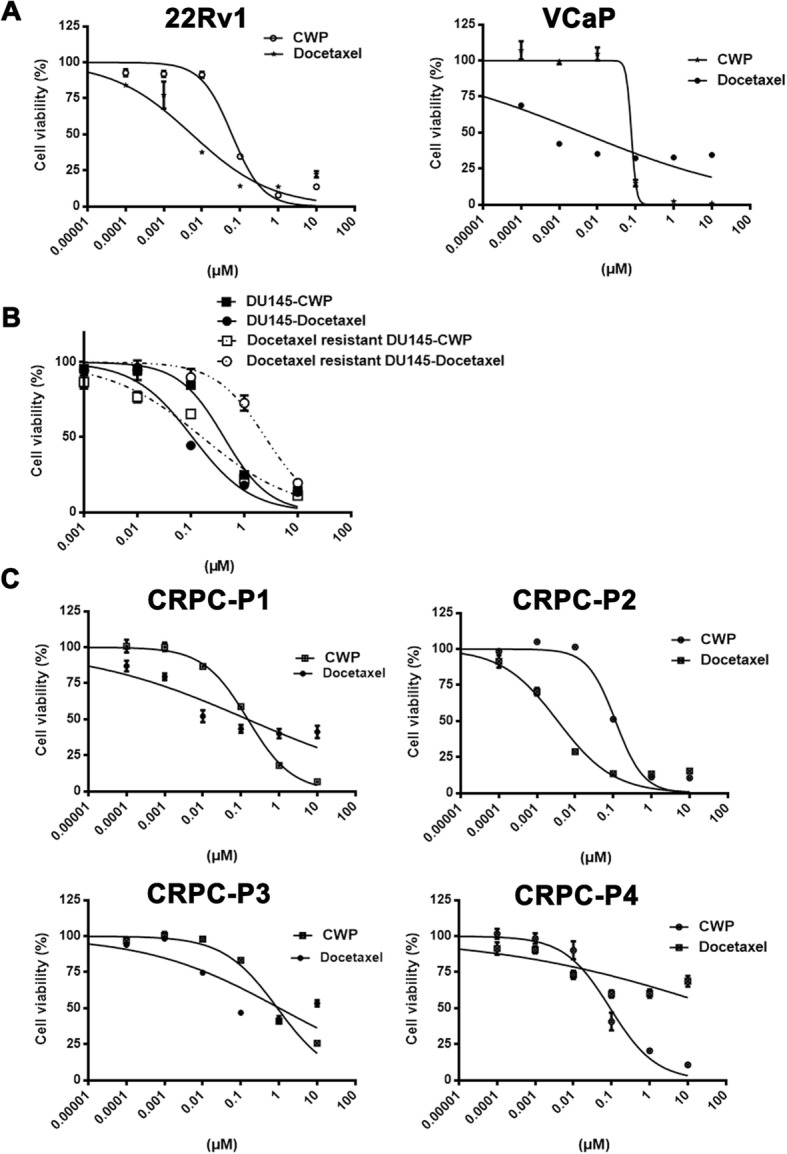
CWP232291 blocks the growth of prostate cancer cells and primary cells derived from castration-resistant prostate cancer patients. **a** 22Rv1 and VCaP cells were exposed to CWP232291 (0–10 μM) and docetaxel (0–10 μM) for 72 h. Cell viability was determined using CellTiter Glo® (means ± SD, *n* = 3). **b** Docetaxel-resistant DU145 and parental DU145 cells were exposed to CWP232291 (0–10 μM) and docetaxel (0–10 μM) for 72 h. Cell viability was determined using CellTiter Glo® (means ± SD, *n* = 3). **c** Primary prostate cancer cells derived from patients with castration-resistant prostate cancer were exposed to CWP232291 (0–10 μM) for 72 h. Cell viability was determined using CellTiter Glo® (means ± SD, *n* = 3)

The effect of CWP232291 was compared with that of the standard of care and tested in the context of castration resistance. 22Rv1 and VCaP cells, which are AR-positive and androgen-independent, were exposed to CWP232291 or docetaxel. CWP232291 inhibited the growth of both 22Rv1 and VCaP cells in a similar manner to docetaxel (Fig. 5a). When tested in docetaxel-resistant prostate cancer cells, CWP232291 showed similar growth inhibition in docetaxel-resistant DU145 cells as in parental DU145 cells (Fig. 5b). Next, CWP232291 was tested in primary prostate cancer cells derived from four CRPC patients (0–10 μM, 72 h) (Fig. 5c). Three patients (P1, P2, and P3) progressed after docetaxel chemotherapy, and one patient (P4) progressed after failure of enzalutamide. As shown in Fig. 5c, CWP232291 showed antitumor activity against all primary prostate cancer cells. Interestingly, CWP232291 suppressed the growth of primary prostate cancer cells from a patient (P4) who progressed after enzalutamide, whereas docetaxel was not effective in these cells.

### CWP232291 inhibits the growth of prostate cancer xenografts in vivo

**Fig. 6:**
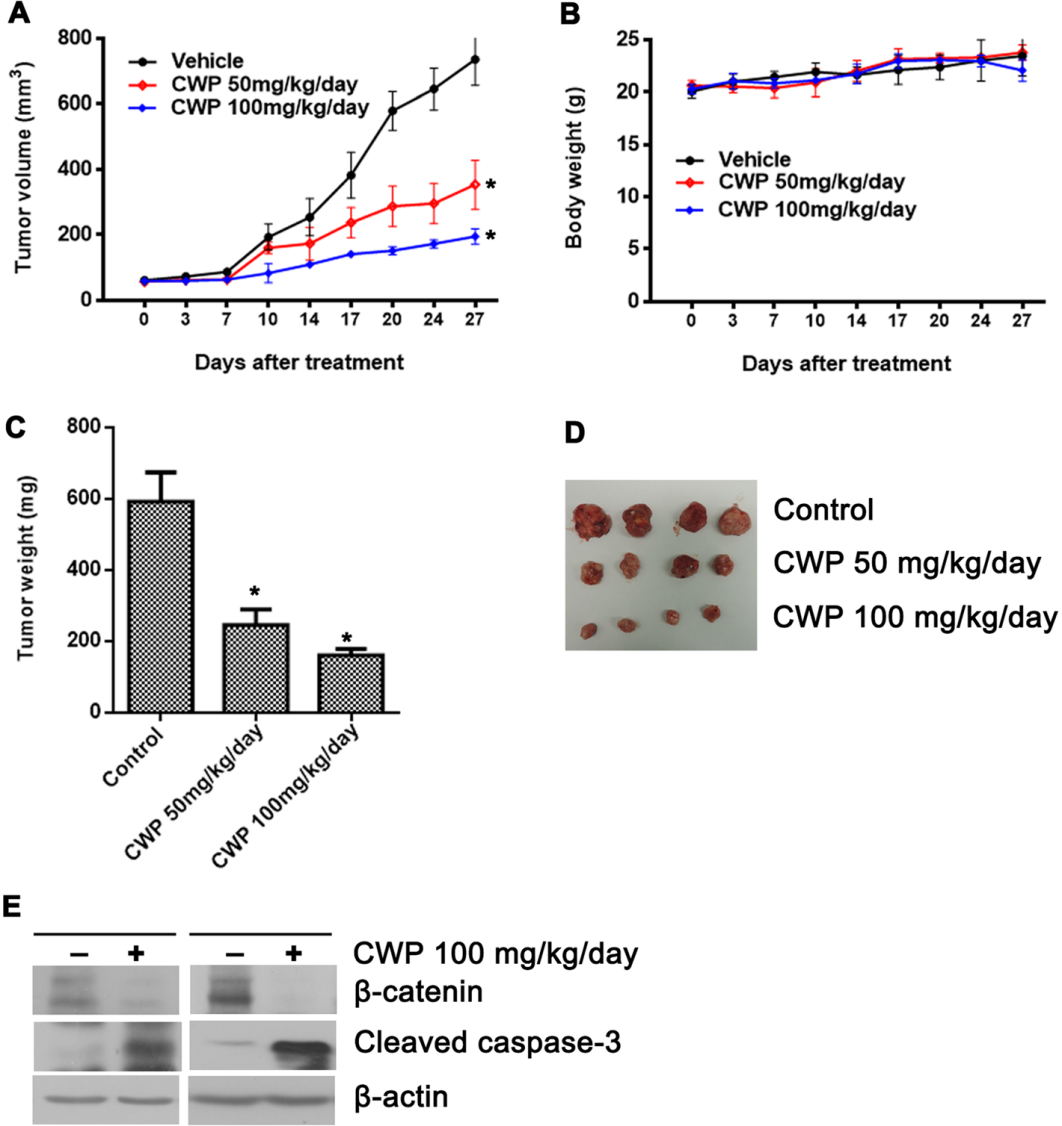
CWP232291 inhibits the growth of prostate cancer xenografts. **a** Mice bearing 22Rv1 tumors were treated with CWP232291 (28 days, 50 mg/kg/day or 100 mg/kg/day). Changes in tumor volume of the 22Rv1 xenografted mice are shown. Results are expressed as the means ± SD of six mice. **b** Body weight of the 22Rv1 xenografted mice is shown. Results are expressed as means ± SD. **c** Tumor weight of the 22Rv1 xenografted mice is shown. Results are expressed as means ± SEM. *P < 0.05 by one-way ANOVA. **d** Representative tumors of the 22Rv1 xenografted mice. **e** Western blot analysis of β-catenin, cleaved caspase-3, and GAPDH in 22Rv1 in two pairs of xenografted tumors. One western blot was randomly selected from the control group and one from the treatment group. Western blots were performed in triplicates and repeated independently three times

The in vivo antitumor activity of CWP232291 was investigated in the 22Rv1 xenograft mouse model (Fig. 6a-e). Mice bearing 22Rv1 tumors were treated with CWP232291 (27 days, 50 mg/kg/day or 100 mg/kg/day). CWP232291 inhibited tumor growth compared with vehicle (Fig. 6a). Tumor growth inhibition reached 52.0% (50 mg/kg/day) and 73.7% (100 mg/kg/day) after 27 days of CWP232291 treatment, while body weight did not change significantly (Fig. 6b). CWP232291 significantly reduced tumor weight (50 mg/kg/day: 245 mg; 100 mg/kg/day: 160 mg; control: 592 mg) after 27 days compared with vehicle (Fig. 6c). Western blotting showed that CWP232291 decreased expression of β-catenin and increased that of cleaved caspase-3 compared with the control, indicating potent growth-inhibiting and proapoptotic effects in vivo in a time- and dose-dependent manner (Fig. 6e).

## Discussion

The present study establishes that the small molecule CWP232291 blocks the growth of CRPC by activating the ER stress pathway and inhibiting β-catenin. CWP232291 induces apoptotic cell death and modulates the WNT pathway, resulting in suppression of β-catenin and AR in prostate cancer cells.

This study shows for the first time that ER stress induction can provide antitumor effects in prostate cancer. The ER is a central organelle involved in cellular homeostasis, stress sensing, and signaling [25]. Various conditions cause an imbalance between protein folding load and capacity in the ER, which is called ER stress. When cancer cells are under ER stress due to intrinsic alterations in cellular metabolism and extrinsic factors in the tumor microenvironment, they respond to this stress by activating the UPR [26]. The UPR can activate adaptive and pro-survival signals or induce apoptotic cell death depending on the severity and duration of ER stress. In the presence of severe and irreparable ER stress, the UPR activates the intrinsic apoptosis pathway [27]. Several studies demonstrated that agents targeting ER stress and the UPR had antiproliferative effects in cancer cells [28–32], suggesting that affecting ER stress pathways may provide novel strategies for cancer therapy. Our results show that CWP232291 triggers ER stress. Upon ER stress induction, the stress sensor PERK is activated, leading to eIF2α phosphorylation. Finally, proapoptotic CHOP is upregulated, resulting in downstream executioner caspase-3-mediated apoptosis.

ER stress is also associated with WNT/β-catenin signaling pathway modulation. Caspase-3-mediated apoptosis can induce degradation of β-catenin [33, 34]. One study reported that severe ER stress can block WNT protein processing and secretion [35]. Mechanistically, CWP disrupts interactions between CBP and β-catenin [36]. Consequently, CWP induces Sam68/CBP complex formation, which alters CBP/β-catenin-dependent transcription to promote apoptosis and differentiation [36]. We observed that LEF/TCF reporter activity in prostate cancer cells increased upon stimulation with WNT3a and decreased with CWP232291.

During the past decade, cancer stem cells have been increasingly considered to play a critical role in tumor development, growth, invasion, and metastasis [37, 38]. In prostate cancer, cancer stem cells are not dependent on androgen for viability, causing progression to CRPC [39]. Given its fundamental role in regulating the proliferation, migration, differentiation, and self-renewal of stem cells, the WNT/β-catenin pathway is considered a new promising cancer stem cell-associated therapeutic target [7, 40].

This study revealed that CWP232291 suppresses the expression of the β-catenin and WNT target gene survivin in prostate cancer. More importantly, CWP232291 also downregulates AR and its splice variants in prostate cancer cells (22Rv1 and LNCaP). WNT pathway modulation may be particularly effective in CRPC because of the synergistic interaction of β-catenin and AR. Synergy between β-catenin and AR has been well documented in previous studies [10–16]. When prostate cancer cells are adapted to a low androgen environment, AR and WNT/β-catenin signaling may reinforce each other to promote androgen-independent growth and progression [41]. Our data show that AR-expressing prostate cancer cells (LNCaP, 22Rv1, and VCaP) are more sensitive to CWP232291 than AR-negative prostate cancer cells (PC3 and DU145). Given the role of AR in CRPC, these results suggest that targeting WNT signaling may be a more effective treatment for AR-positive than AR-negative CRPC through the concurrent disruption of β-catenin and AR signaling.

There is evidence that activation of WNT/β-catenin signaling induces chemoresistance in several solid tumors [42–44]. The clinical implication of these findings is that WNT/β-catenin signaling modulation may be effective in chemoresistant cancer [45, 46]. Our finding that CWP232291 shows the same antitumor activity in docetaxel-resistant and docetaxel-sensitive DU145 cells supports that hypothesis. These results suggest that CWP232291 may have therapeutic potential as a second-line treatment after chemotherapy failure in CRPC.

Despite academic and industrial research efforts in WNT/β-catenin signaling in several cancers, no WNT/β-catenin inhibitor is yet approved for human use. Several small molecules that inhibit WNT/β-catenin signaling are in preclinical or early clinical phase of development [46]. CWP232291 is currently being tested in phase 1A/1B clinical trials in patients with acute myeloid leukemia and multiple myeloma [17–19]. To the best of our knowledge, the effect of CWP232291 in prostate cancer has not been investigated. A major concern with the therapeutic use of WNT/β-catenin inhibition is safety since the WNT/β-catenin signaling cascade is also critical in normal somatic stem cell homeostasis and tissue maintenance [47]. CWP232291 has shown a favorable safety profile in clinical trials. In the interim results of ongoing trials, CWP232291 showed early evidence of efficacy and grade 3/4 adverse events were rare [17, 18].

## Conclusions

In summary, this study provides the first demonstration that the small molecule WNT/β-catenin inhibitor CWP232291 blocks the growth of prostate cancer cells in vitro, ex vivo, and in vivo. CWP232291 induces ER stress and upregulates the proapoptotic transcription factor CHOP. Mechanistically, ER stress is also associated with WNT pathway modulation. CWP232291 suppresses the expression of β-catenin and AR in CRPC. Our results provide preclinical evidence of therapeutic efficacy for the novel WNT/β-catenin inhibitor CWP232291 in CRPC.

## Additional file

Additional file 1: **Figure S1.** Plasma concentration of the active metabolite (CWP232204) after a single intravenous injection of CWP232291 into nude mice (doses: 25, 50, and 100 mg/kg). (A): Logarithmic scale, (B): Normal scale. **Figure S2.** Cells were exposed to CWP232291 for 24 h and WNT3a and then stained with DAPI (blue) or β-catenin (green). Images were captured using a fluorescence microscope (Olympus). **Figure S3.** Cells were exposed to 0–10 μM CWP232291 for 72 h. The cell cytotoxicity assay was performed using the Cytotoxicity Detection Kit (Sigma Aldrich, St. Louis, MO). **Figure S4.** Reporter assay for androgen-response elements after treatment with or without DHT and CWP232291 (LNCaP; 100nM, 22Rv1 60nM). **Figure S5.** 22Rv1 cells were androgen-deprived for 48h and then treated with vehicle or 10 nM DHT with or without 60 nM CWP232291 for 24 h. Chromatin immunoprecipitation analyses demonstrated that CWP232291 inhibits β-catenin occupancy of the TCF-binding site within the AR promoter. **Table S1.** CWP232291 pharmacokinetic variables. (DOCX 912 kb)

## Data Availability

The dataset used and/or analyzed during the current study are available from the corresponding author on reasonable request.

## Abbreviations

ADT: Androgen deprivation therapy
AR: Androgen receptor
ATF6: Activating transcription factor 6
CHOP: C/EBP-homologous protein
CRPC: Castration-resistant prostate cancer
DAPI: 4′,6-diamidino-2-phenylindole
DHT: Dihydrotestosterone
FBS: Fetal bovine serum
IRE1: Inositol-requiring kinase 1
LDH: Lactate dehydrogenase
LEF: Lymphoid enhancer-binding factor
PARP: Poly (ADP-ribose) polymerase
PERK: Protein kinase RNA-like ER kinase
RT-PCR: Real-time polymerase chain reaction
TCF: Transcription factor
UPR: Unfolded protein response
ZVAD-FMK: Z-valine-alanine-aspartate-fluoromethylketone
