# Correction to: The small molecule WNT/β-catenin inhibitor CWP232291 blocks the growth of castration-resistant prostate cancer by activating the endoplasmic reticulum stress pathway

**DOI:** 10.1186/s13046-019-1451-1

**Published:** 2019-10-31

**Authors:** Sahyun Pak, Sejun Park, Yunlim Kim, Jung-Hyuck Park, Chan-Hee Park, Kyoung-June Lee, Choung-soo Kim, Hanjong Ahn

**Affiliations:** 1 Department of Urology, Center for Urologic Cancer, National Cancer Center, Goyang, South Korea; 2 Department of Urology, University of Ulsan College of Medicine, Ulsan University Hospital, Ulsan, South Korea; 3 Department of Urology, University of Ulsan College of Medicine, Asan Medical Center, Seoul, South Korea; 4 Asan Institute for Life Science, Asan Medical Center, Seoul, South Korea; 5 Drug Discovery Center, JW Pharmaceutical Corporation, Seoul, South Korea; 6 JW Creagene Corporation, Seongnam, South Korea

**Correction to: J Exp Clin Cancer Res (2019) 38: 342**

**https://doi.org/10.1186/s13046-019-1342-5**

In the original publication of this article [1], there are mistakes in Fig. 4d. The corrected Fig. 4 should be:

**Fig. 4:**
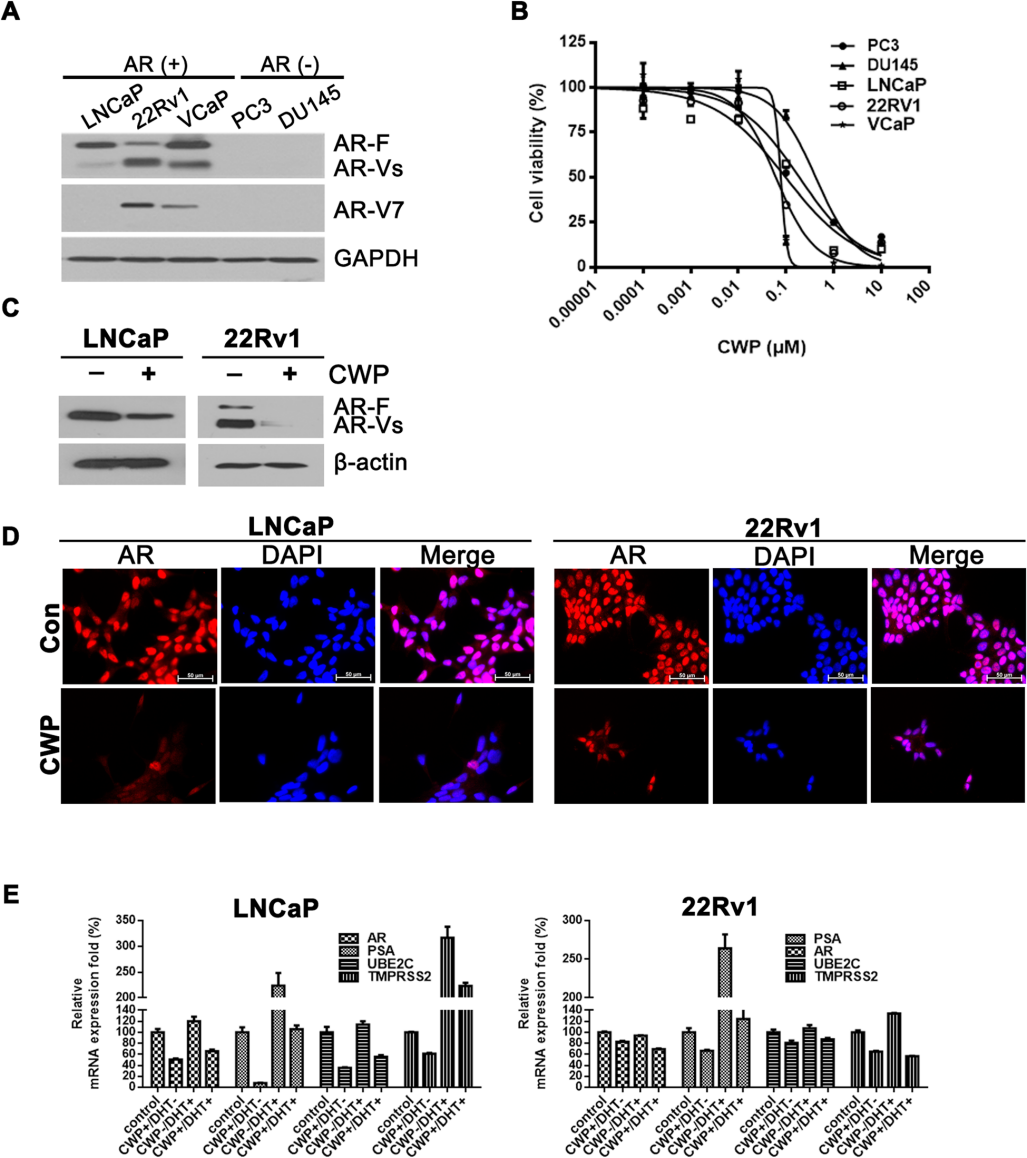
.
