# Supplementary material for: The small molecule WNT/β-catenin inhibitor CWP232291 blocks the growth of castration-resistant prostate cancer by activating the endoplasmic reticulum stress pathway
Source: J Exp Clin Cancer Res. 2019 Aug 6;38:342. doi: 10.1186/s13046-019-1342-5 (PMC6685284; doi:10.1186/s13046-019-1342-5)

**Additional file 1**

**Table of Contents**

1. Methods

2. Figures

Figure S1. Plasma concentration of the active metabolite (CWP232204) after a single intravenous injection of CWP232291 into nude mice (doses: 25, 50, and 100 mg/kg). (A) : Logarithmic scale, (B) : Normal scale

Figure S2. Cells were exposed to CWP232291 for 24 h and WNT3a and then stained with DAPI (blue) or β-catenin (green). Images were captured using a fluorescence microscope (Olympus).

Figure S3. Cells were exposed to 0–10 μM CWP232291 for 72 h. The cell cytotoxicity assay was performed using the Cytotoxicity Detection Kit.

Figure S4. Reporter assay for androgen-response elements after treatment with or without DHT and CWP232291 (LNCaP; 100nM, 22Rv1 60nM).

Figure S5. 22Rv1 cells were androgen-deprived for 48h and then treated with vehicle or 10 nM DHT with or without 60 nM CWP232291 for 24 h. Chromatin immunoprecipitation analyses demonstrated that CWP232291 inhibits β-catenin occupancy of the TCF-binding site within the AR promoter

3. Table

Table S1. CWP232291 pharmacokinetic variables.

**Methods**

**ARE reporter assay**

The ARE reporter assay was determined using the androgen receptor reporter kit from Qiagen (Toronto, Ontario, Canada). LNCaP and 22Rv1 cells were transfected with a mixture of an inducible androgen receptor-responsive firefly luciferase construct and a construct constitutively expressing Renilla luciferase (40:1 ratio). The AR-responsive luciferase construct encodes the firefly luciferase reporter gene under the control of a CMV promoter and tandem repeats of the AR transcriptional response element. Twenty-four hours after cells were transfected with the inducible ARE luciferase construct in the presence or absence of DHT (10nM) and CWP232291 (LNCaP; 100nM, 22Rv1 60nM), the luciferase activities of the cells were measured with a Dual-Luciferase reporter assay kit (Promega, Madison, WI).

**Chromatin immunoprecipitation assay**

22Rv1 cells were androgen-deprived for 48h and then treated with vehicle or 10 nM DHT with or without 60 nM CWP232291 for 24 h. Cells were fixed in 1% formaldehyde at room temperature and the cross-linking reaction was quenched with 125 mM glycine. After cell lysis, chromatin was fragmented into 150–500 bp fragments using a sonicator (power, 3 W; sonication time, 20 s; no. of repeats, 3) and protein-DNA complexes were immuno-captured by mixing magnetic bead-coated anti-β-catenin antibody (10 μg; Santa Cruz Biotechnology, Dallas, TX) with the fragmented chromatin on a rotator at 4°C overnight. After washing and reversal of crosslinks, the IP DNA and input DNAs were purified and amplified by PCR. Relative enrichment was calculated as a percentage of 1% input normalized to IgG. The forward (CAAAATTGAGCGCCTATGTG) and reverse (TTGCTCTAGGAACCCTCAGC) primers of the TCF-binding site on the AR promoter were used.

Figure S1. Plasma concentration of the active metabolite (CWP232204) after a single intravenous injection of CWP232291 into nude mice (doses: 25, 50, and 100 mg/kg). (A) : Logarithmic scale, (B) : Normal scale


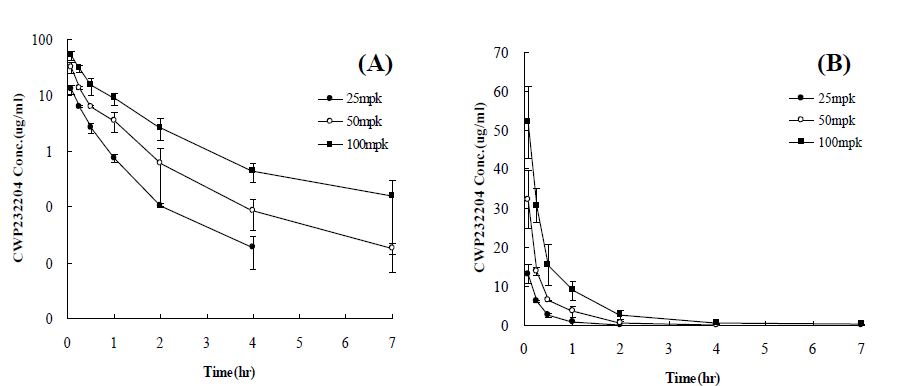


Figure S2. Cells were exposed to CWP232291 for 24 h and WNT3a and then stained with DAPI (blue) or β-catenin (green). Images were captured using a fluorescence microscope (Olympus).


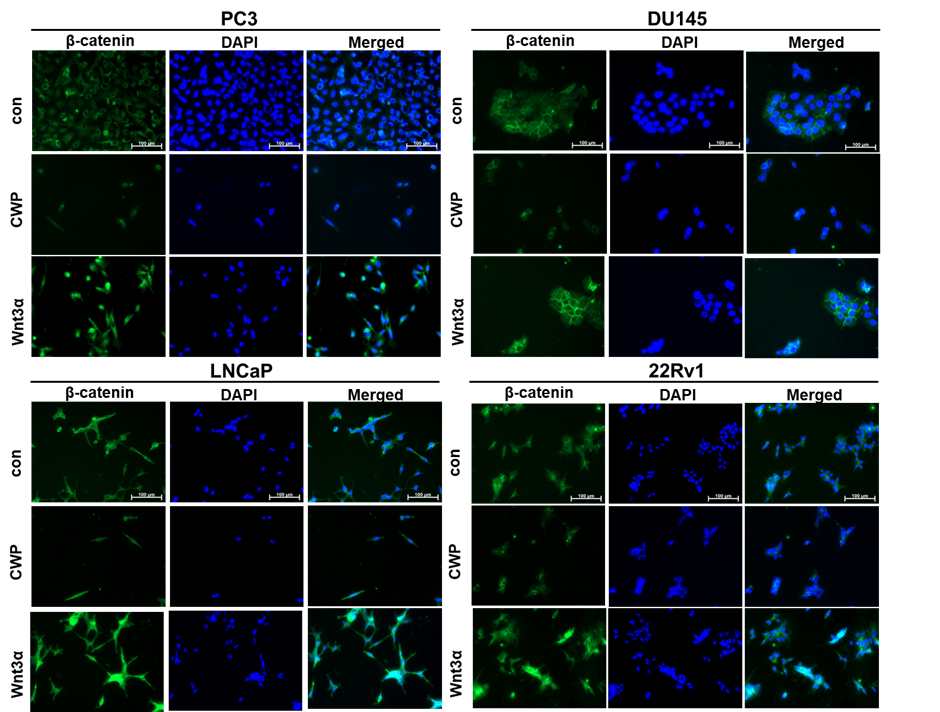


Figure S3. Cells were exposed to 0–10 μM CWP232291 for 72 h. The cell cytotoxicity assay was performed using the Cytotoxicity Detection Kit.


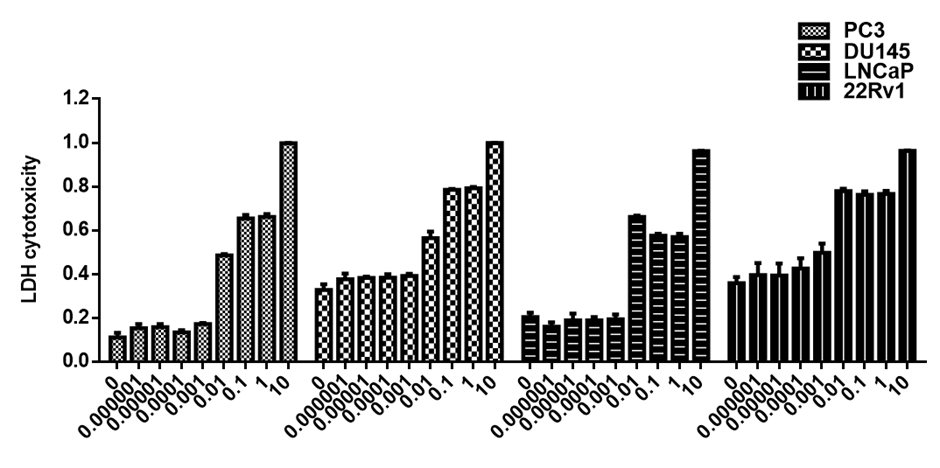


Figure S4. Reporter assay for androgen-response elements after treatment with or without DHT and CWP232291 (LNCaP; 100nM, 22Rv1 60nM).

Figure S5. 22Rv1 cells were androgen-deprived for 48h and then treated with vehicle or 10 nM DHT with or without 60 nM CWP232291 for 24 h. Chromatin immunoprecipitation analyses demonstrated that CWP232291 inhibits β-catenin occupancy of the TCF-binding site within the AR promoter.

Table S1. CWP232291 pharmacokinetic variables.


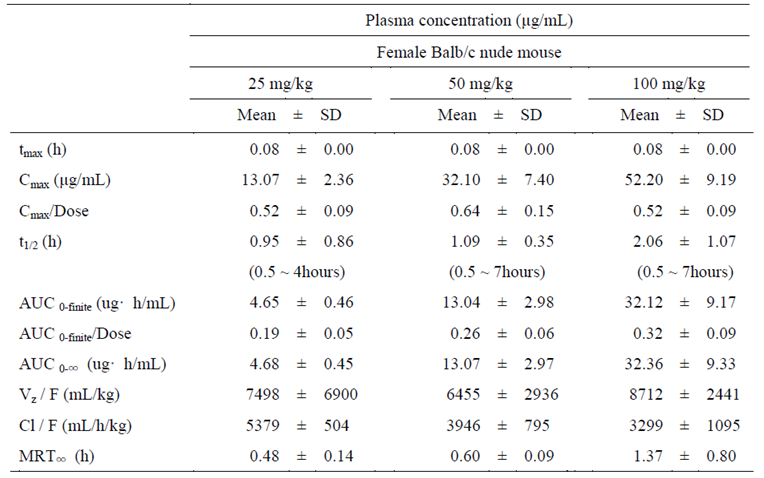

Supplement: Supplementary file 1 — Figure S1. Plasma concentration of the active metabolite (CWP232204) after a single intravenous injection of CWP232291 into nude mice (doses: 25, 50, and 100 mg/kg). (A): Logarithmic scale, (B): Normal scale. Figure S2. Cells were exposed to CWP232291 for 24 h and WNT3a and then stained with DAPI (blue) or β-catenin (green). Images were captured using a fluorescence microscope (Olympus). Figure S3. Cells were exposed to 0–10 μM CWP232291 for 72 h. The cell cytotoxicity assay was performed using the Cytotoxicity Detection Kit (Sigma Aldrich, St. Louis, MO). Figure S4. Reporter assay for androgen-response elements after treatment with or without DHT and CWP232291 (LNCaP; 100nM, 22Rv1 60nM). Figure S5. 22Rv1 cells were androgen-deprived for 48h and then treated with vehicle or 10 nM DHT with or without 60 nM CWP232291 for 24 h. Chromatin immunoprecipitation analyses demonstrated that CWP232291 inhibits β-catenin occupancy of the TCF-binding site within the AR promoter. Table S1. CWP232291 pharmacokinetic variables. (DOCX 912 kb) [file 13046_2019_1342_MOESM1_ESM.docx]
